# A prospective clinical study evaluating short-term changes in body composition and quality of life after gastrectomy in elderly patients receiving postoperative exercise and nutritional therapies

**DOI:** 10.1186/s12893-023-02086-4

**Published:** 2023-06-29

**Authors:** Yasunori Nishida, Mai Tokunaga, Akira Kameyama, Masatake Miyamoto, Seijiro Yoshifuku, Kotaro Sasahara, Noriaki Otagiri, Katsunori Tauchi

**Affiliations:** 1 Department of Surgery, Aizawa Hospital, 2-5-1 Honjou Matsumoto, Nagano, 390-8510 Japan; 2 Rehabilitation Center, Aizawa Hospital, 2-5-1 Honjou Matsumoto, Nagano, 390-8510 Japan

**Keywords:** Gastric cancer, Body composition, Elderly, BCAA, Rehabilitation

## Abstract

**Background:**

Muscle mass loss after gastrectomy is associated with a negative impact on quality of life (QOL) and long-term prognosis following gastric cancer treatment, especially in elderly patients. We conducted a prospective study to examine short-term changes in body composition and QOL after gastrectomy in elderly patients with gastric cancer who received exercise and nutritional therapies.

**Methods:**

Patients over aged 65 years of age who underwent gastrectomies for gastric cancer were enrolled in our study. Patients received exercise and nutritional therapies with branched-chain amino acid (BCAA)-rich supplements during 1 month after surgery. Body composition was evaluated using InBody S10 before surgery, and at 1 week and 1 month postoperatively. Other variables including QOL status (EQ-5D-5 L), serum albumin level, hand grip strength, and gait speed were evaluated at the same time.

**Results:**

Eighteen patients were analyzed. The mean loss of skeletal muscle mass index (SMI) was 4.6% (1 week) and 2.1% (1 month) compared to the preoperative period. QOL scores showed almost the same degree of recovery at 1 month after gastrectomy as preoperative scores. Serum albumin levels, hand grip strength, and gait speed decreased at 1 week and then increased at 1 month after surgery, similar to the changes seen in SMI.

**Conclusions:**

Multidisciplinary approaches play key role in the surgical treatment of elderly patients. Postoperative exercise and nutritional therapies with BCAA-rich supplements may benefit elderly patients after gastrectomy by reducing loss of SMI and decreases in QOL.

**Trial registration:**

UMIN Clinical Trials Registry; UMIN000034374 (registration date: 10/10/2018).

**Supplementary Information:**

The online version contains supplementary material available at 10.1186/s12893-023-02086-4.

## Background

Gastrectomy plays a key role in gastric cancer treatments. However, depression of stomach function after gastrectomy often induces severe weight loss, muscle mass loss, and nutritional insufficiency, which are associated with decreased quality of life (QOL) [1–3]. Recently, muscle mass loss after gastrectomy has been recognized as an important prognostic factor in patients with gastric cancer [4, 5].　Aoyama et al. reported that the loss of lean body mass at 1 week and at 1 month after surgery was 2.6% and 6.0%, respectively, in elderly patients (≥ 80 years) and 3.5% and 4.9% in non-elderly patients [6]. Gastrectomy impacts more adversely in elderly patients compared to younger patients, due to attenuation of muscle mass and strength with aging.

Elderly people constitute the majority of the world’s population and are growing rapidly in number [7]. Several interventions, including exercise and nutritional therapies before and after gastrectomy, have been implemented to prevent weight loss and muscle mass loss [8–10]. However, few studies of perioperative interventions in elderly patients with gastric cancer have focused on muscle mass, function, and QOL.

The purpose of this study was to examine changes in body composition, function, and QOL status after gastrectomy in elderly patients with gastric cancer who received exercise and nutritional therapies with branched-chain amino acid (BCAA)-rich supplements.

## Patients and methods

### Study patients

This prospective study was approved by the institutional review board of the Aizawa Hospital (#2018-004). Written informed consent was obtained from all patients. This study was registered in the University Hospital Medical Information Network Trials Registry (UMIN000034374). The eligibility criteria included: (1) patients aged 65 years or older, (2) patients in whom distal or total gastrectomy for gastric cancer was performed with curative intent, and (3) patients with an Eastern Cooperative Oncology Group performance status of 0–1. Exclusion criteria included patients who underwent neoadjuvant chemotherapy and those with severe or uncontrolled liver and/or kidney disease. Patients were checked for eligibility and were registered before surgery.

### Perioperative management including postoperative exercise and nutritional therapies with protein (BCAA)-rich supplements

Patients underwent distal or total gastrectomy with regional lymph node dissection according to the Japanese Gastric Cancer Treatment Guidelines [11]. Laparoscopic surgery was conducted for clinical stage I cancer resectable by distal gastrectomy according to the guidelines [11].

Patients started oral intake on postoperative day (POD) 2, initiated with water and an oral nutritional supplement. They began to eat solid food (rice gruel, 400 kcal/day) on POD 3, and advanced gradually to regular food intake (900 kcal/day) on POD 7 over three steps. Dietitians provided individual nutritional counseling before and after gastrectomy.

Patients received an organized rehabilitation program for 1 month after surgery. In this study, protein (BCAA)-rich supplements (REHADAYS; Otsuka Pharmaceutical Factory, Inc., Tokushima, Japan) were added to the program to enhance the effects of rehabilitation. Each 125mL pack of the protein (BCAA)-rich supplement contains 160 kcal, 11.0 g protein, and 2300 mg L-leucine. During hospitalization, patients underwent rehabilitation with physical therapists. Patients went through a stir-up regimen which included deep breathing, coughing, positioning, mobilization, and pain management on POD 1 and 2. After sufficient mobilization, the patients initiated exercise regimens focused on moderate-intensity walking and resistance training. Walking for more than 5000 steps per day was recommended. Resistance training included three sets of ten repetitions of calf raises and squats. After hospital discharge, the patients performed the exercise regimen at home. A protein (BCAA)-rich supplement was recommended to be taken just after the exercises (1 pack per day).

Hospital discharge was recommended around POD 9 with adequate food intake and activity. A self-completed questionnaire was used to evaluate patients’ achievements of the exercise regimen and dietary intake.

### Assessment

Body composition was evaluated using InBody S10 body water analyzer (InBody Japan, Tokyo, Japan) before surgery as well as 1 week and 1 month after surgery. Changes in body composition including skeletal muscle mass index (SMI), lean body mass, and fat mass, was evaluated. In addition, body weight, serum albumin level, hand grip strength, gait speed, and QOL were evaluated at the same time. Grip strength was evaluated with a handgrip dynamometer for assessment of muscle strength. Gait speed was evaluated using a 6-meter gait speed test. For QOL evaluations, a 5-level EQ-5D questionnaire (EQ-5D-5 L) developed by the EuroQol Group was used [12, 13] (Registration number: 26529). The QOL index was calculated using EQ-5D-5 L tariffs published by Shiroiwa et al. [14].

Data collected from the patients included demographics, comorbidities, laboratory data, surgical characteristics, postoperative complications, postoperative hospital stay, and pathological findings. The American Society of Anesthesiologists (ASA) classification score [15] was obtained from the preoperative anesthesia evaluation. Comorbid conditions were scored using the Charlson comorbidity index [16]. Pathological tumor staging was based on the tumor-node-metastasis (TNM) stage classified by the Union for International Cancer Control (UICC) (8th edition) [17].

### Statistical analysis

All analyses were performed using the IBM SPSS Statistics 21 software package (SPSS Inc.). The Mann-Whitney *U* test was used to compare continuous variables, and chi-square analysis was used to compare categorical variables. Statistical significance was set at *p* < 0.05.

## Results

### Patient characteristics and perioperative outcomes

Eighteen patients over 65 years of age who underwent gastrectomies for gastric cancer between January 2019 and February 2020 were enrolled in this study. The demographic and clinical characteristics of patients in this study are shown in Table 1. The mean patient age was 75.3 years, and 12 patients (66.7%) were male. Half of the patients underwent total gastrectomy, and laparoscopic surgery was performed in 6 patients (33.3%). Postoperative complications (Clavien-Dindo classification grade ≥ grade II) occurred in 5 patients, and there were no fatalities in this study. One patient with anastomostic leakage had an extended hospital stay; however, most of patients left the hospital as scheduled on POD 9. Mean protein (BCAA)-rich nutritional supplements intake was 57.7% (Additional File Fig. 1).

**Table 1:** Patient characteristics and perioperative outcomes

Variables	*n* = 18
**Preoperative characteristics**	
Age, years	75.3 (4.78)
Gender (male/female), n	12/6
BMI, kg/m^2^	22.3 (3.6)
SMI, kg/m^2^	6.9 (1.2)
ASA score (I/II), n	2/16
Charlson comorbidity index score*	6 (5–8)
Gait speed (m/s)	1.20 (0.17)
Handgrip strength (kg)	32.5 (8.1)
Serum albumin (g/dL)	4.1 (0.4)
**Operative variables**	
Type of gastrectomy (TG/DG), n	9/9
Operative approach (open/laparoscopic), n	12/6
Reconstructive procedure (TG, Roux-en-Y/DG, Roux-en-Y/DG, Billroth I), n	9/7/2
Operation time, min*	262 (154–351)
Blood loss, ml*	85 (10–480)
**Postoperative outcomes and pathological finding**	
Complications (≥ C-D grade II)	5
Postoperative hospital stay, days*	9.0 (9–82)
Pathological stage (I/II/III), n	10/5/2

***BMI*** body mass index, ***SMI*** skeletal muscle mass index, ***ASA*** American society of anesthesiologists, ***TG*** total gastrectomy, ***DG*** distal gastrectomy, ***C-D*** Clavien-Dindo classicication

Continuous parameters are presented as mean (standard deviation) or median (range)*

### Short-term changes in body composition and physical performance

**Fig. 1:**
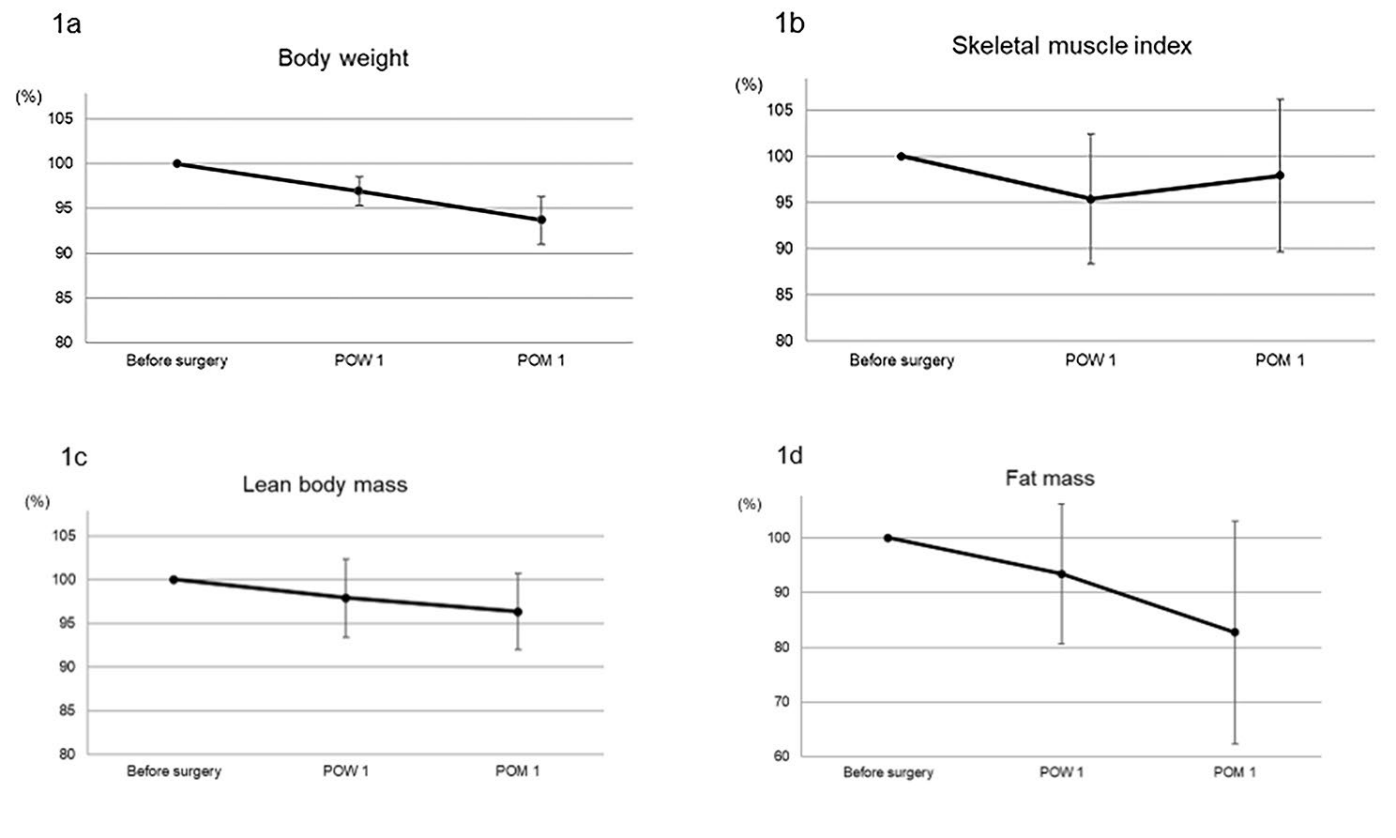
Changes in body composition before surgery, at 1 week after gastrectomy, and 1 month after gastrectomy. (**a**) Body weight. (**b**) Skeletal muscle index. (**c**) Lean body mass. (**d**) Fat mass *POW* postoperative week, *POM* postoperative month

The mean percentage of body weight loss at 1week and 1 month after surgery compared to presurgical data were 3.1% and 6.3%, respectively (Fig. 1a). Mean changes in SMI were 4.6% and 2.1%, decreasing at 1 week and increasing at 1 month after surgery (Fig. 1b). Mean changes in lean body mass were 2.1% and 3.7% (Fig. 1c). In addition, mean changes in fat mass were 6.6% and 17.3% (Fig. 1d). Serum albumin levels, hand grip strength, and gait speed decreased at 1 week and then increased at 1 month after surgery (Table 2), similar to the changes seen in SMI.

**Table 2:** Short-term changes in body composition and physical/nutritional performance

	Before surgery	POW 1	POM 1
Body weight (kg)	57.9 (11.2)	56.1 (10.6)	54.2 (10.3)
BMI (kg/m^2^)	22.3 (3.6)	21.7 (3.5)	21.0 (3.6)
SMI (kg/m^2^)	6.9 (1.2)	6.6 (1.1)	6.6 (1.2)
Gait speed (m/s)	1.20 (0.17)	1.04 (0.22)	1.19 (0.37)
Handgrip strength (kg)	32.5 (8.1)	32.0 (8.4)	32.6 (8.1)
Serum albumin (g/dL)	4.1 (0.4)	3.2 (0.4)	3.9 (0.4)

*POW* postoperative week, *POM* postoperative month, *BMI* body mass index, *SMI* skeletal muscle mass index

Continuous parameters are presented as mean (standard deviation)

Body composition changes stratified by surgical approach (open/laparoscopic) and procedure (distal/total gastrectomy) are shown in Additional File Fig. 2 and 3. The open approach and total gastrectomy tended to induced greater depletion of body composition than other conditions.

### QOL evaluations

**Fig. 2:**
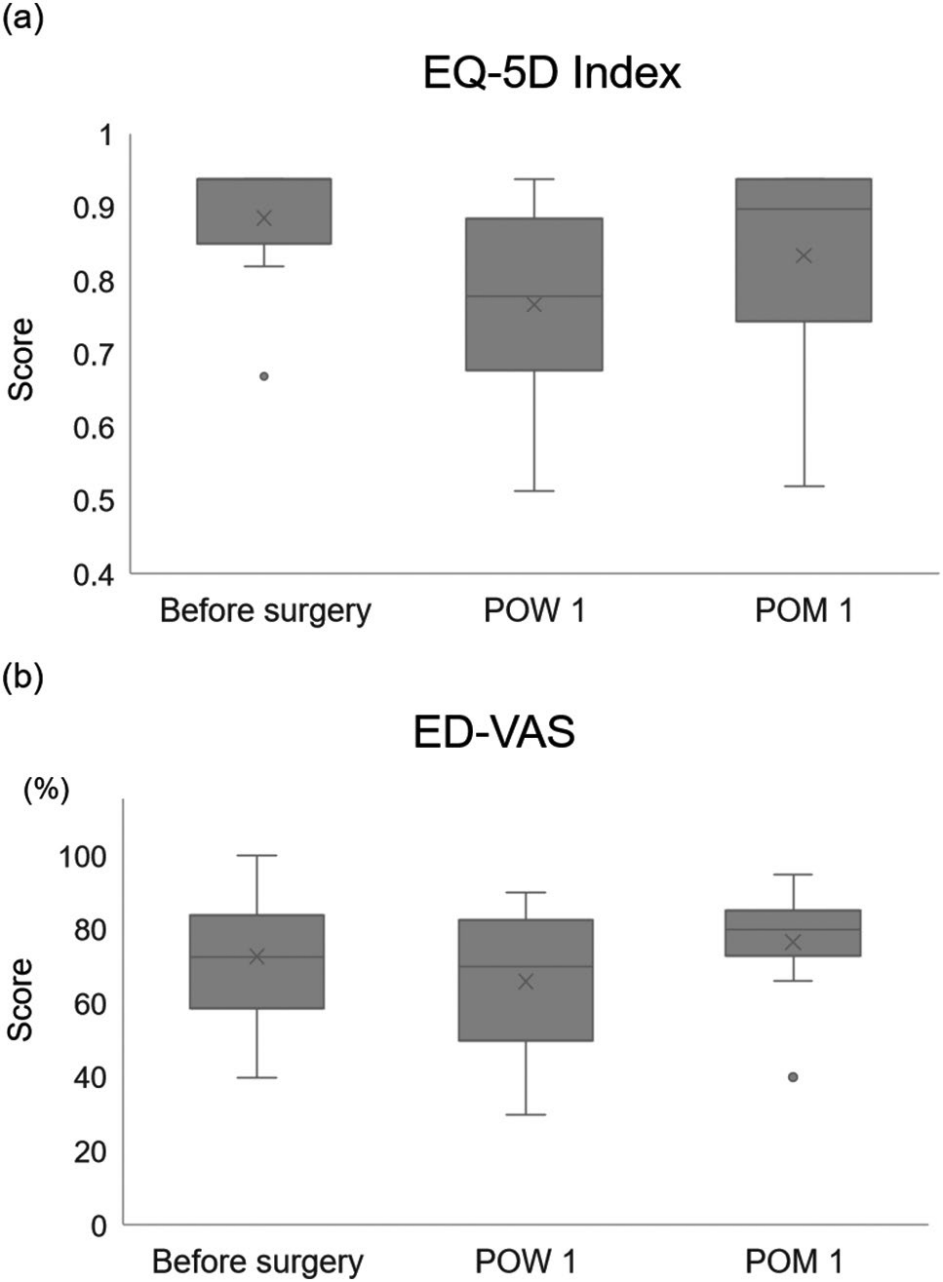
QOL evaluation using 5-level EQ-5D questionnaire (EQ-5D-5 L). Boxplots of health-related quality of life scores before surgery, 1 week after surgery, and 1 month after surgery. (**a**) EQ-5D index scores. (**b**) ED-VAS scores *QOL* quality of life, *POW* postoperative week, *POM* postoperative month, *VAS* visual analogue scale

Mean EQ-5D index scores were 0.8855 before surgery, 0.7669 1 week after surgery, and 0.8338 1 month after surgery (Fig. 2a). The QOL VAS is a vertical visual analogue scale that asks patients to rate their general health from 0 to 100. Mean ED-VAS scores were 72.6 before surgery, 65.9 at 1 week, and 76.6 at 1 month after surgery (Fig. 2b). QOL parameters recovered shortly after gastrectomy compared to presurgical levels.

## Discussion

Muscle mass loss is associated with negative impact on QOL and long-term prognosis following gastric cancer treatment [1–3], with higher impact on elderly patients. In this prospective study, we examined to examine short-term changes in body composition and QOL after gastrectomy in elderly gastric cancer patients receiving postoperative exercise and nutritional therapies with protein (BCAA)-rich nutritional supplements. The results of this study showed that muscle mass and SMI changed relatively little in elderly patients 1 month after gastrectomy. In addition, physical function and QOL recovered shortly after gastrectomy.

Loss of muscle and body mass within a few months after gastrectomy has been reported to be 5–15% [6, 18]. Aoyama et al. reported that loss of lean body mass at 1 week and 1 month after surgery were 2.6% and 6.0%, respectively, in elderly patients (≥ 80) and 3.5% and 4.9% in non-elderly patients [6]. Additionally, recent studies have shown that these losses influence the continuity of adjuvant chemotherapy and long-term prognosis [4, 5, 19]. In elderly patients, loss of muscle mass and body weight after gastrectomy adversely affects daily activities and QOL. Elderly patients require a different level of perioperative management than non-elderly patients. Increasing prevalence of gastric cancer makes it important to advance perioperative management of elderly patients with gastric cancer. Abdiev et al. reported that body composition changes were significant during the first month after gastrectomy [18]. Therefore, preventing muscle muss loss and QOL decrease during the short-term after gastrectomy is important. This study focused on the short-term changes in body composition after gastrectomy in elderly patients with gastric cancer. In this study, the mean SMI loss was 4.6% (1 week) and 2.1% (1 month), and loss in body weight were 3.1% and 6.3% at 1 week and 1 month after surgery, respectively. Compared with previous studies [6, 18], the exercise and nutritional therapies in this study may help to prevent body composition changes after gastrectomy.

QOL can be impaired after gastrectomy [20–22]. Van den Wieden et al. reported QOL changes using the EQ-5D in their randomized controlled trial [20]. Van den Boorn et al. also showed that the largest decline in global health status was observed in the first month after gastrectomy, and the status returned to baseline 12 months after surgery [22]. In our study, QOL scores showed almost the same degree of recovery at 1 month after gastrectomy as preoperative scores. Indeed, there was a significant age-specific difference in QOL status after gastrectomy [21]. The results of our study, which included patients over 65 years of age, were similar to those of a previous study that included all-age patients [20]. The multimodal interventions in this study may help to prevent QOL decrease after gastrectomy.

Several exercise and/or nutritional therapies can be administered before and after gastrectomy to prevent loss of weight and muscle mass [8–10]. Yamamoto et al. evaluated the effects of preoperative nutritional intervention with exercise in 22 sarcopenic patients with gastric cancer who were aged ≥ 65 years [9]. The study concluded that preoperative nutritional and exercise intervention may reduce sarcopenia in older adult sarcopenic patients with gastric cancer. Regarding exercise interventions after gastrectomy, Cho et al. evaluated the safety and feasibility of a postoperative recovery exercise program (without nutritional intervention) in gastric cancer patients undergoing minimally invasive gastrectomies [10]. While the population in their study was younger and the patients underwent minimally invasive surgery, muscle volume was preserved after completing the program compared to the preoperative period [10]. Based on these findings, perioperative exercise interventions have the potential to enhance physical function and QOL [23]. However, few studies of postoperative interventions (including exercise and nutritional therapies) in elderly patients with gastric cancer focused on muscle mass, function, and QOL. Therefore, the results of our study will be useful for postoperative management of elderly patients with gastric cancer.

BCAA supplementation has been reported to be effective in improving body composition by correcting amino acid imbalance [24]. Some systematic reviews [25–27] have shown the efficacy of a combination of exercise and protein supplementation in improving muscle strength and physical function in frail older persons. In this study, we used protein (BCAA)-rich nutritional supplements to enhance the effects of exercise and nutritional therapies. The mean intake of protein (BCAA)-rich nutritional supplements in this study was similar to that in a previous study using other nutritional supplements [28]. As this study was not conducted to evaluate the effects of protein (BCAA)-rich nutritional supplements, we are unable to discuss the influence of BCAA on the outcomes of body composition changes. However, protein (BCAA)-rich nutritional supplements may potentially impact postoperative outcomes by increasing total calorie intake and improving body composition.

This study had some limitations. First, this was a single-institutional, non-randomized study with one arm and a small sample size. Second, long-term outcomes were not assessed in this study. However, body weight and muscle mass decreased significantly within a short-time after gastrectomy, which impact greatly on gastric cancer treatment and daily life. Therefore, this study assessed the short-term outcomes of changes in body composition and QOL. Third, it is unclear from this study which factors affect body composition and QOL changes. Further studies are needed to assess the factors that influence patients after gastrectomy.

Multidisciplinary approaches play key role in the surgical treatment of elderly patients. Postoperative exercise and nutritional therapies with protein (BCAA)-rich supplements may benefit elderly patients undergoing gastrectomy by reducing loss of SMI and QOL.

## Electronic supplementary material

Below is the link to the electronic supplementary material.

**Additional File Fig 1**: Compliance with protein (BCAA)-rich nutritional supplement intake.

**Additional File Fig 2**: Changes in body composition before surgery, at 1 week after gastrectomy, and 1 month after gastrectomy, stratified by surgical approach (open/laparoscopic).

**Additional File Fig 3**: Changes in body composition before surgery, at 1 week after gastrectomy, and 1 month after gastrectomy, stratified by surgical approach (distal/total gastrectomy).

## Data Availability

Please contact the corresponding author for data requests.

## Abbreviations

QOL: Quality of life
BCAA: branched-chain amino acid
POD: Postoperative day
SMI: skeletal muscle mass index
EQ-5D-5L: 5-level EQ-5D
ASA: American Society of Anesthesiologists
TNM: Tumor-node-metastasis
UICC: Union for International Cancer Control
