# Supplementary material for: A prospective clinical study evaluating short-term changes in body composition and quality of life after gastrectomy in elderly patients receiving postoperative exercise and nutritional therapies
Source: BMC Surg. 2023 Jun 29;23:181. doi: 10.1186/s12893-023-02086-4 (PMC10311715; doi:10.1186/s12893-023-02086-4)
Supplement: Supplementary file 1 — Additional File Fig 1: Compliance with protein (BCAA)-rich nutritional supplement intake. [file 12893_2023_2086_MOESM1_ESM.docx]

**Supplementary Figure 1** Compliance with protein (BCAA)-rich nutritional supplement intake.

**
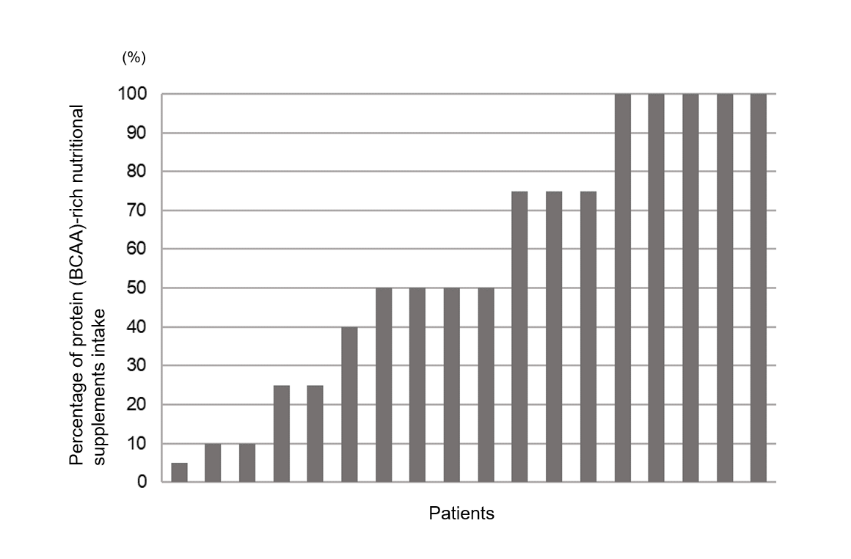
**

*BCAA* branched-chain amino acid
