# Supplementary material for: A prospective clinical study evaluating short-term changes in body composition and quality of life after gastrectomy in elderly patients receiving postoperative exercise and nutritional therapies
Source: BMC Surg. 2023 Jun 29;23:181. doi: 10.1186/s12893-023-02086-4 (PMC10311715; doi:10.1186/s12893-023-02086-4)
Supplement: Supplementary file 2 — Additional File Fig 2: Changes in body composition before surgery, at 1 week after gastrectomy, and 1 month after gastrectomy, stratified by surgical approach (open/laparoscopic). [file 12893_2023_2086_MOESM2_ESM.docx]

**Supplementary Figure 2** Changes in body composition before surgery, at 1 week after gastrectomy, and 1 month after gastrectomy, stratified by surgical approach (open/laparoscopic).


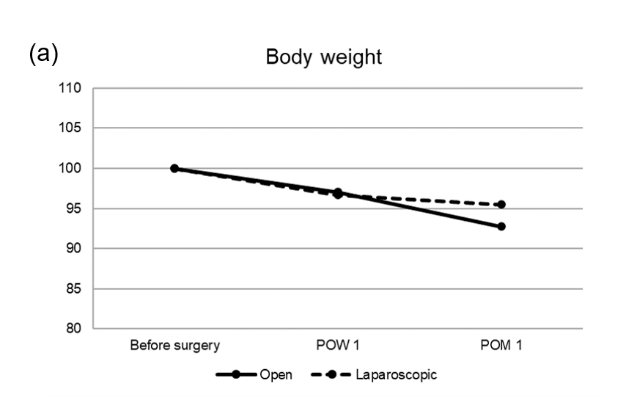

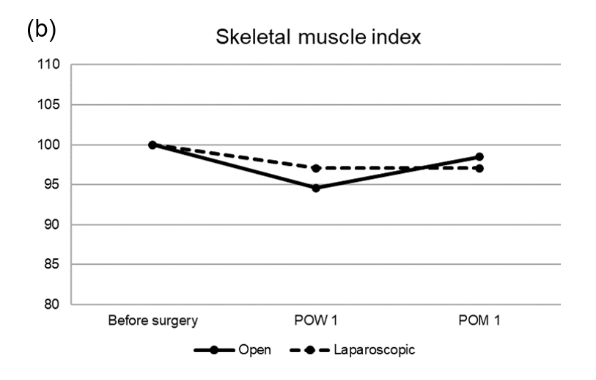


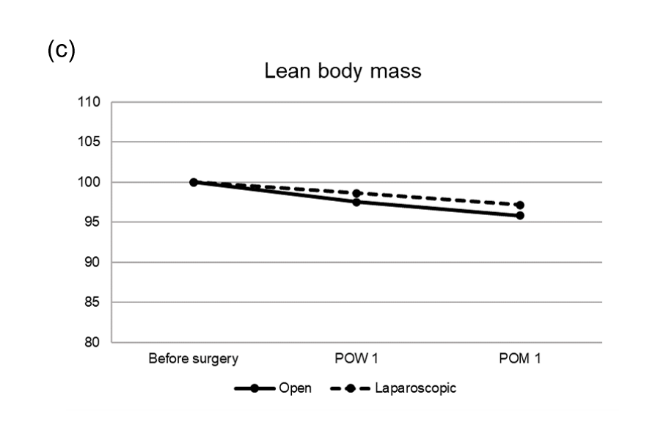

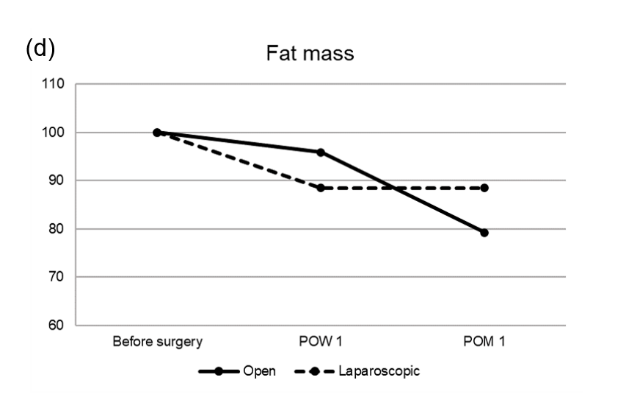


(a) Body weight. (b) Skeletal muscle index. (c) Lean body mass. (d) Fat mass.

*POW* postoperative week, *POM* postoperative month
